# Thirty-Day Mortality and Morbidity in COVID-19 Positive vs. COVID-19 Negative Individuals and vs. Individuals Tested for Influenza A/B: A Population-Based Study

**DOI:** 10.3389/fmed.2020.598272

**Published:** 2020-11-30

**Authors:** Vardan Nersesjan, Moshgan Amiri, Hanne K. Christensen, Michael E. Benros, Daniel Kondziella

**Affiliations:** ^1^Department of Neurology, Rigshospitalet, Copenhagen University Hospital, Copenhagen, Denmark; ^2^Department of Neurology, Bispebjerg Hospital, Copenhagen University Hospital, Copenhagen, Denmark; ^3^Department of Clinical Medicine, Faculty of Health and Medical Sciences, University of Copenhagen, Copenhagen, Denmark; ^4^Copenhagen Research Centre for Mental Health - CORE, Mental Health Centre Copenhagen, Copenhagen University Hospital, Hellerup, Denmark; ^5^Department of Immunology and Microbiology, Faculty of Health and Medical Sciences, University of Copenhagen, Copenhagen, Denmark

**Keywords:** COVID-19, SARS-CoV-2, coronavirus, ischemic heart disease, morbidity, mortality, neurology, psychiatry

## Abstract

**Background:** As of October 2020, COVID-19 has caused 1,000,000 deaths worldwide. However, large-scale studies of COVID-19 mortality and new-onset comorbidity compared to individuals tested negative for COVID-19 and individuals tested for influenza A/B are lacking. We investigated COVID-19 30-day mortality and new-onset comorbidity compared to individuals with negative COVID-19 test results and individuals tested for influenza A/B.

**Methods and findings:** This population-based cohort study utilized electronic health records covering roughly half (*n* = 2,647,229) of Denmark's population, with nationwide linkage of microbiology test results and death records. All individuals ≥18 years tested for COVID-19 and individuals tested for influenza A/B were followed from 11/2017 to 06/2020. Main outcome was 30-day mortality after a test for either COVID-19 or influenza. Secondary outcomes were major comorbidity diagnoses 30-days after the test for either COVID-19 or influenza A/B. In total, 224,639 individuals were tested for COVID-19. To enhance comparability, we stratified the population for in- and outpatient status at the time of testing. Among inpatients positive for COVID-19, 356 of 1,657 (21%) died within 30 days, which was a 3.0 to 3.1-fold increased 30-day mortality rate, when compared to influenza and COVID-19-negative inpatients (all *p* < 0.001). For outpatients, 128 of 6,263 (2%) COVID-19-positive patients died within 30 days, which was a 5.5 to 6.9-fold increased mortality rate compared to individuals tested negative for COVID-19 or individuals tested positive or negative for influenza, respectively (all *p* < 0.001). Compared to hospitalized patients with influenza A/B, new-onset ischemic stroke, diabetes and nephropathy occurred more frequently in inpatients with COVID-19 (all *p* < 0.05).

**Conclusions:** In this population-based study comparing COVID-19 positive with COVID-19 negative individuals and individuals tested for influenza, COVID-19 was associated with increased rates of major systemic and vascular comorbidity and substantially higher mortality. Results should be interpreted with caution because of differences in test strategies for COVID-19 and influenza, use of aggregated data, the limited 30-day follow-up and the possibility for changing mortality rates as the pandemic unfolds. However, the true COVID-19 mortality may even be higher than the stated 3.0 to 5.5-fold increase, owing to more extensive testing for COVID-19.

## Introduction

COVID-19 has led to a worldwide healthcare crisis with >30,000,000 confirmed infected people, resulting in 1,000,000 deaths as of October 2020 (1, 2). Governmental initiatives including lockdowns and social distancing are aiming to restrict the spread of the virus. Yet, critical voices (3) have argued the socioeconomic consequences may be unjustified given that little is known about how the pandemic compares with annual influenza epidemics in terms of mortality and morbidity. According to the WHO seasonal influenza A/B may result in 290,000–650,000 deaths worldwide annually (4, 5). Substantially higher mortality rates for COVID-19 will result in even more adverse impact on global health without strict preventive measures. However, large-scale studies including follow-up of individuals tested for COVID-19 and influenza A/B from the same cohort are lacking.

Of further concern, COVID-19 might not only be a respiratory disease but a multi-organ disorder because of the wide expression of the angiotensin-converting enzyme-2 receptor to which SARS-Cov-2 binds (6), leading among others to thromboembolic complications (7), severe inflammatory responses (8), and possibly diabetes (9). Neurological and psychiatric complications will likely constitute a major health burden as well (10, 11). But how COVID-19 morbidity compares to similarly severe influenza morbidity is equally poorly understood.

Here, for the first time, we utilized population-based electronic health records (EHR) from Denmark linked with nationwide databases on test results for infections and death records, to investigate mortality in people with COVID-19 compared to people with influenza and to people tested negative for COVID-19. For secondary outcomes we estimated COVID-19-associated new-onset comorbidity, including cardiovascular, neurological and psychiatric events, compared to influenza and individuals tested COVID-19-negative. Analyses were stratified according to age, sex and in- and outpatient status. We hypothesized that COVID-19 would be associated with higher mortality and increased rates of novel comorbidities compared to influenza A/B.

## Materials and Methods

This retrospective Danish study was based on EHR covering two well-defined administrative regions: Capital Region (i.e., Greater Copenhagen and Bornholm) and Region Zealand, comprising roughly 50% of the Danish population. Denmark has an almost exclusively public health care sector based on catchment areas.

### Registers and Study Population

The EHR system of the Capital and Zealand Regions, which is called EPIC (version 2019, Verona, Wisconsin, USA), consists of data from all hospital contacts in these regions. From implementation in 2016 to June 30, 2020, 2,647,229 individuals were registered. Diagnoses are defined according to ICD-10 (12). Registration of death in the EHR is synchronized with the Danish national population registry, updated every 24 h. Accuracy of test results for influenza and SARS-CoV-2 virus is ensured by synchronization of EPIC with the nationwide Danish Microbiology Database (13). All individuals ≥18 years tested for COVID-19 between March 1-June 1, 2020, and all individuals tested for influenza A/B between November 1, 2017-June 1, 2020, were followed for mortality and new-onset comorbidities 30-days after the test until June 30, 2020. Included individuals in this study were hospitalized patients who were tested for COVID-19 or influenza during admission (from now on referred to as *inpatients*); and non-hospitalized patients screened during ambulatory visit, as well as healthy individuals screened in hospital-based testing facilities created for the purpose of screening the general population (from now on referred to as *outpatients*).

### Assessment of COVID-19 and Influenza Test Results

#### COVID-19

All individuals tested for COVID-19 during March 1-June 1, 2020 with laboratory tests CORONAVIRUS 2019-NCOV and/or CORONAVIRUS SARS-COV-2 RNA via nasal, pharyngeal and/or tracheal samples with reverse-transcriptase-polymerase-chain reaction (RT-PCR) assays were included. These specific tests cover all performed COVID-19 tests in the catchment areas and are available from the Danish Microbiology Database (13).

#### Influenza A/B

We included all individuals tested for influenza A/B during November 1, 2017 to March 1, 2020, using 9 different RT-PCR laboratory tests (Supplementary Table 1), covering all available influenza tests based on nasal, pharyngeal and/or tracheal samples.

### Outcome Measures

#### Primary Outcome Measures

Thirty-day mortality among the group of inidividuals tested positive for COVID-19, compared to 30-day mortality of the group of individuals with COVID-19-negative tests. Additional comparisons were made to the group of individuals tested influenza-positive or influenza-negative.

#### Secondary Outcome Measures

*N*ew-onset (i.e., 30 days after COVID-19 or influenza test) comorbidity diagnoses, including neurological, psychiatric and cardiovascular disease, pulmonary embolism, venous thrombosis, renal failure, diabetes and rheumatoid arthritis, in all populations. ICD-10 codes are listed in Supplementary Table 1.

### Data Collection, Statistical Analysis, and Ethics

Anonymized retrospective aggregate-level data on sex, age, prior comorbidities and population mortality 30 days after test results were extracted for individuals ≥18 years for each groups, using the EPIC Slicer-Dicer function. For search strategies see Supplementary Table 1. As individuals could be tested multiple times, individuals were only included in the COVID-19-negative, respectively, influenza-negative populations, when all their tests had been negative. Individuals tested for influenza during March 1-June 1, 2020 (i.e., FLU-19) were included for sensitivity analysis (see below). To avoid overlap, we removed COVID-19-positive individuals from the FLU-19 group.

Main analysis was the relative risk (RR) of mortality rates 30 days after a test, in the overall populations and stratified according to in- and outpatient status, sex, and age. Secondary analysis was RR of cumulative 30 days post-test incidence of new-onset comorbidities, after exclusion of individuals who already had the investigated comorbidity before the test. We compared COVID-19 positive with COVID-19-negative and influenza-positive individuals. To validate mortality data, absolute mortality rates extracted from electronic health records (EPIC) were compared with official Danish statistics numbers (Supplementary Table 2). Sensitivity analysis was conducted by comparing individuals ≥18 years with a positive or negative influenza test from the same time period as the COVID-19 population, i.e., March 1-June 1, 2020 (FLU-19), in order to investigate the possible influence of the COVID-19 pandemic, including lockdown and social distancing measures, on mortality rates in individuals tested for influenza. Chi-squared statistics were used to calculate odds ratio (OR), RR and 95% confidence intervals (CI) using SPSS (version 25; IBM, Armonk, NY, USA). Two-sided *p* ≤ 0.05 was considered significant.

The Ethics Committee of the Capital Region of Denmark waives approval for register-based studies on aggregated anonymized data (Section 14.2 of the Committee Act. 2; http://www.nvk.dk/english). Use of anonymized aggregate-level data was approved by the Danish Data Protection Agency. Results from ≤5 patients were displayed as “ ≤5” to ensure data privacy.

## Results

**Figure 1:**
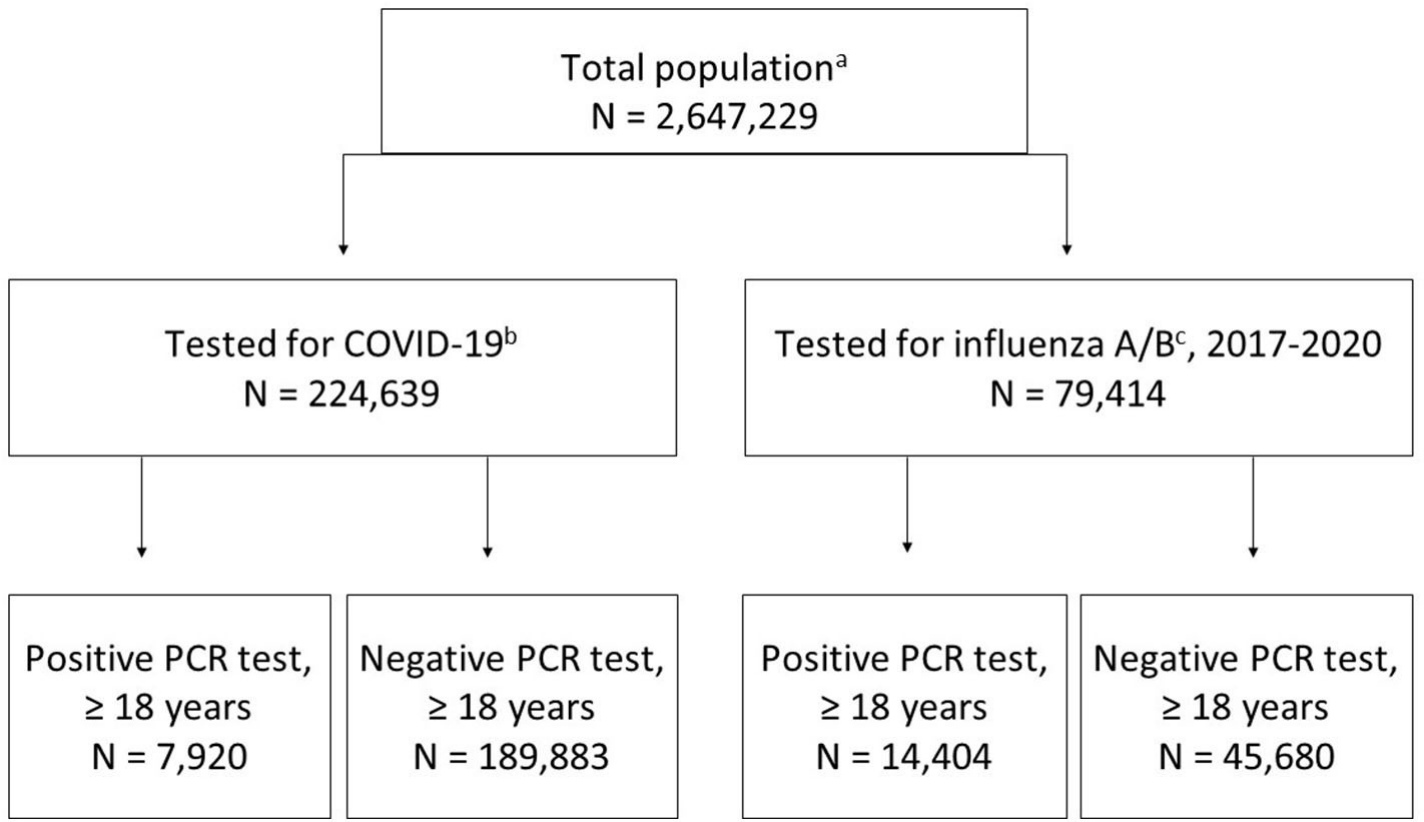
Flow chart of study populations. (a) total population in EHR registered per June 30, 2020; (b) tested between March 1-June 1, 2020; (c) tested between November 1, 2017-March 1, 2020.

A total of 224,639 individuals of any age were tested for SARS-CoV-2 between March 1-June 1, 2020; positive results were found in 7,920 individuals ≥18 years (i.e., our case population). A negative COVID-19 test occurred in 189,883 individuals ≥18 years. Between November 1, 2017-March 1, 2020, we identified 79,414 individuals, who were tested for influenza A/B. Positive results were found in 14,404 individuals aged ≥18 years. Negative influenza A/B tests were identified in 45,680 individuals ≥18 years (Figure 1). Demographics are displayed in Table 1 and Supplementary Tables 3–5. The proportion of inpatients at the time of COVID-19 or influenza tests was lower in the COVID-19-positive (20.9%) and the COVID-19-negative (16.6%) populations compared to influenza-positive (50%) and influenza-negative (57.7%) populations. We therefore analyzed mortality and comorbidities both in the overall populations and stratified according to in- and outpatient status, sex, and age.

**Table 1:** Demographics and prior comorbidities among individuals tested for COVID-19 or influenza as in- or outpatient.

	**Inpatients**			**Outpatients**		
	**COVID-19 positive** **(*N* = 1,657)**	**COVID-negative** **(*N* = 31,483)**	**Influenza-positive** **(*N* = 7,200)**	**COVID-19-positive** **(*N* = 6,263)**	**COVID-negative** **(*N* = 158,400)**	**Influenza-positive** **(*N* = 7,204)**
**Age – years, no. (%)**						
Mean, years	65	60	66	47	48	49
18–39	182 (11.0)	7,456 (23.7)^‡^	931 (12.9)	2,310 (36.9)	54,659 (34.5)^‡^	2,390 (33.2)^‡^
40–59	427 (25.8)	6,790 (21.6)^‡^	1,413 (19.6)^‡^	2,509 (40.1)	62,701 (39.6)	2,637 (36.6)^‡^
60–80	636 (38.4)	11,261 (35.8)^‡^	2,876 (39.9)	1,043 (16.7)	33,166 (20.9)^‡^	1,762 24.4)^‡^
> 80	412 (24.9)	5,976 (19.0)^‡^	1,980 (27.5)	401 (6.4)	7,874 (5.0)^‡^	415 (5.8)
**Sex (%)**						
Women	737 (44.5)	17,099 (54.3)^‡^	3,844 (53.4)^‡^	3,931 (62.8)	99,364 (62.7)	4,272 (59.3)^‡^
**Prior medical diagnoses - no. (%)**^**#**^						
Neurological, any	275 (16.6)	4,998 (15.9)	1,025 (14.2)	505 (8.1)	14,074 (8.9)^‡^	474 (6.6)^‡^
Cerebrovascular, any	141 (8.5)	2,819 (9.0)	485 (6.7)	145 (2.3)	3,698 (2.3)	125 (1.7)^‡^
Ischemic stroke incl. TIA	78 (4.7)	1,739 (5.5)	165 (2.3)^‡^	85 (1.4)	2,302 (1.5)	130 (1.8)^‡^
Psychiatric, any	202 (12.2)	6,581 (20.9)^‡^	821 (11.4)	383 (6.1)	12,264 (7.7)^‡^	305 (4.2)^‡^
Ischemic heart disease	147 (8.9)	2,631 (8.4)	547 (7.6)	90 (1.4)	3,719 (2.3)^‡^	155 (2.2)^‡^
Heart failure	100 (6.0)	1,861 (5.9)	422 (5.9)	45 (0.7)	1,581 (1.0)^‡^	68 (0.9)
Diabetes	199 (12.0)	3,291 (10.5)^‡^	827 (11.5)	209 (3.3)	4,903 (3.1)	255 (3.5)
Chronic lower respiratory disease	182 (11.9)	3,943 (12.5)	1,362 (18.9)^‡^	223 (3.6)	7,934 (5.0)^‡^	366 (5.1)^‡^
Obesity	46 (2.8)	1,296 (4.1)^‡^	167 (2.3)	132 (2.1)	3,929 (2.5)	141 (2.1)

*COVID-19, positive COVID-19 PCR test between March 1-June 1, 2020; influenza, positive influenza A/B PCR test between November 1, 2017 to March 1, 2020; COVID-neg, negative COVID-19 PCR test between March 1-June 1, 2020. Each patient was followed for a total of 30 days from positive test until end of follow-up or death*.

‡ *Indicates statistically significant difference (p < 0.05) compared to COVID-19 populations*.

# *Established medical diagnoses, registered in the medical files, prior to testing for COVID-19 or influenza. TIA, transitory ischemic attack*.

### Primary Outcome: Mortality

#### Overall Mortality Rates

Overall 30-day mortality in COVID-19-positive individuals was 484 of 7,920 (6.1%), whereas 30-day mortality for COVID-19-negative individuals was 2,654 of 189,883 (1.4%), corresponding to an increased mortality by RR 4.37 (95% CI = 3.98–4.80).

#### Mortality Rates of Inpatients Tested for COVID-19 and/or Influenza

**Figure 2:**
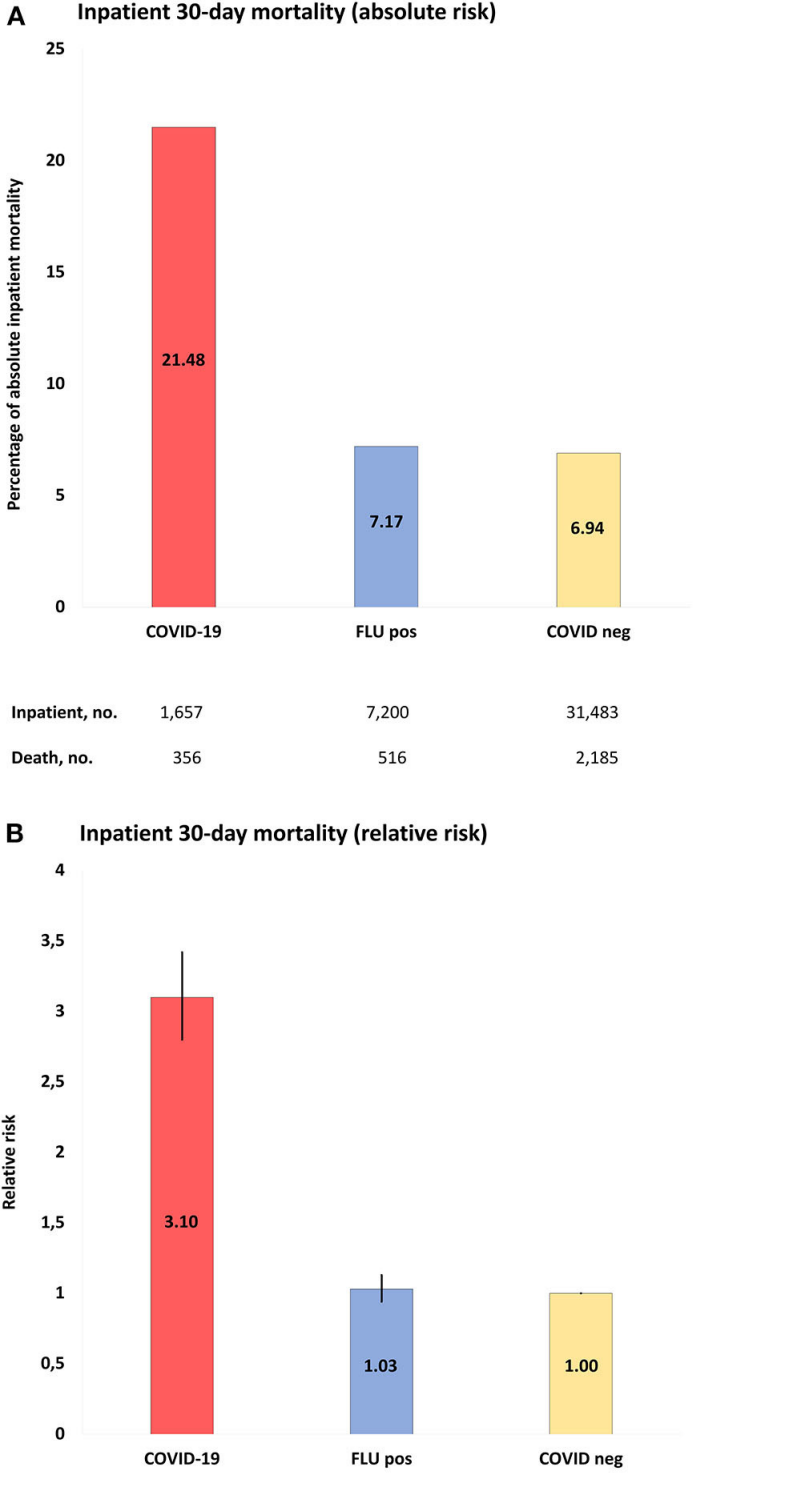
**(A)**, Absolute risk with 95% CI of COVID-19 inpatient 30-day mortality, when compared to populations of the study: COVID-19, positive COVID-19 test between March 1-June 1, 2020; FLU pos, positive influenza A/B test between November 1, 2017-March 1, 2020; COVID-neg, negative COVID-19 test between March 1-June 1, 2020. **(B)**, RR with 95% CI of inpatient mortality of study populations compared to COVID-19 negative population as reference.

Thirty-day mortality for hospitalized COVID-19 patients ≥18 years was 356 of 1,657 (21.5%), which was higher than in COVID-19-negative individuals (30-day mortality 2,185/31,483; 6.9%; *p* < 0.001) (Figure 2, Table 2, and Supplementary Tables 6–9). The corresponding numbers for individuals tested positive for influenza were 516/7,200 (7.2%) and for influenza-negative individuals 2,873/26,366 (11%). Mortality for COVID-19-positive inpatients was increased by RR 3.10 (95% CI = 2.80–3.42) compared to COVID-19-negative patients, and by RR 3.00 (95% CI = 2.65–3.39) and RR 1.97 (95% CI = 1.79–2.18) compared to influenza-positive, respectively, influenza-negative inpatients (all *p* < 0.001).

**Table 2:** Relative risk of 30-day mortality after a COVID-19 or influenza test among in- or outpatients.

	**Inpatients**			**Outpatients**		
	**Total (N)**	**Death (N)**	***RR (95% CI)***	**Total (N)**	**Death (N)**	***RR (95% CI)***
**COVID-19-positive vs. COVID-negative**						
Overall	1,657 vs. 31,483	356 vs. 2,185	**3.10** (2.80–3.42)^†^	6,263 vs. 158,400	128 vs. 469	**6.90** (5.69–8.38)^†^
Female	738 vs. 17,134	143 vs. 1,027	**3.23** (2.76–3.79)^†^	3,937 vs. 99,512	75 vs. 261	**7.26** (5.63–9.37)^†^
Male	919 vs. 14,349	213 vs. 1,158	**2.87** (2.52–3.27)^†^	2,326 vs. 58,888	53 vs. 208	**6.45** (4.79–8.70)^†^
18–39 years	182 vs. 7,456	≤5 vs. 15	N/A	2,310 vs. 54,659	≤5 vs. ≤5	N/A
40–59 years	427 vs. 6,790	16 vs. 158	1.61 (0.97-2.67)	2,509 vs. 62,701	≤5 vs. 20	N/A
60–80 years	636 vs. 11,261	150 vs. 1,004	**2.65** (2.27–3.08)^†^	1,043 vs. 33,166	33 vs. 161	**6.52** (4.50–9.43)^†^
> 80 years	412 vs. 5,976	190 vs. 1,008	**2.73** (2.42–3.08)^†^	401 vs. 7,874	92 vs. 288	**6.27** (5.07–7.76)^†^
**COVID-19 positive vs. FLU-positive**						
Overall	1,657 vs. 7,200	356 vs. 516	**3.00** (2.65–3.39)^†^	6,263 vs. 7,204	128 vs. 27	**5.45** (3.61–8.25)^†^
Female	738 vs. 3,851	143 vs. 240	**3.11** (2.57–3.77)^†^	3,937 vs. 4,284	75 vs. 17	**4.80** (2.84–8.11)^†^
Male	919 vs. 3,349	213 vs. 276	**2.81** (2.39–3.31)^†^	2,326 vs. 2,920	53 vs. 10	**6.65** (3.39–13.05)^†^
18–39 years	182 vs. 931	≤5 vs. ≤5	N/A	2,310 vs. 2,390	≤5 vs. ≤5	N/A
40–59 years	427 vs. 1,413	16 vs. 26	**2.04** (1.10–3.76)^‡^	2,509 vs. 2,638	≤5 vs. ≤5	N/A
60–80 years	636 vs. 2,876	150 vs. 214	**3.17** (2.62–3.83)^†^	1,043 vs. 1,761	33 vs. ≤5	N/A
> 80 years	412 vs. 1,980	190 vs. 271	**3.37** (2.89–3.92)^†^	401 vs. 415	92 vs. 20	**4.76** (2.30–7.57)^†^
**COVID-19 positive vs. FLU-negative**						
Overall	1,657 vs. 26,366	356 vs. 2,873	**1.97** (1.79–2.18)^†^	6,263 vs. 19,314	128 vs. 129	**3.06** (2.40–3.90)^†^
Female	738 vs. 13,456	143 vs. 1,275	**2.05** (1.75–2.39)^†^	3,937 vs. 11,773	75 vs. 60	**3.74** (2.67–5.24)^†^
Male	919 vs. 12,910	213 vs. 1,598	**1.87** (1.65–2.12)^†^	2,326 vs. 7,541	53 vs. 69	**2.49** (1.75–3.55)^†^
18–39 years	182 vs. 3,867	≤5 vs. 40	N/A	2,310 vs. 6,664	≤5 vs. ≤5	N/A
40–59 years	427 vs. 4,757	16 vs. 224	0.79 (0.48–1.31)	2,509 vs. 6,454	≤5 vs. 6	N/A
60–80 years	636 vs. 10,939	150 vs. 1,307	**1.97** (1.70–2.30)^†^	1,043 vs. 4,963	33 vs. 51	**3.08** (2.00–4.75)^†^
> 80 years	412 vs. 6,803	190 vs. 1,302	**2.41** (2.15–2.70)^†^	401 vs. 1,233	92 vs. 71	**3.98** (2.98–5.32)^†^

*COVID-19, positive COVID-19 test between March 1-June 1, 2020; COVID-neg, negative COVID-19 test between March 1-June 1, 2020; FLU positive, positive influenza A/B test between November 1, 2017-March 1, 2020; FLU negative, negative influenza A/B test between November 1, 2017-March 1, 2020. Each included patient was followed for a total of 30 days from positive test until end of follow-up or death. N/A, not applicable due to low number of patients (N ≤ 5)*.

† *p < 0.001*;

‡ *p < 0 .05. The bold values indicate statistically significant values*.

When mortality rates were stratified according to age, 30-day mortality rates for hospitalized COVID-19 patients were 16/427 (3.7%, age 40–59 years), 150/636 (23.6%, 60–80 years) and 190/412 (46%, >80 years). The corresponding numbers for COVID-19-negative individuals were 158/6,790 (2.3%), 1,004/11,261 (8.9%), and 1,008/5,976 (16.9%) and for influenza-positive individuals 26/1,413 (1.8%), 214/2,876 (7.4%), and 271/1,980 (13.7%), respectively. Mortality for COVID-19-positive inpatients was significantly increased with age 60–80 years (RR = 2.65; 95% CI = 2.27–3.08) and >80 years (RR = 3.17; 95% CI = 2.62–3.83), when compared to COVID-19-negative individuals (RR 2.73; 95% CI = 2.42–3.08) and influenza-positive individuals (RR 3.37 (95% CI = 2.89–3.92). When mortality rates were stratified according to sex, 30-day mortality rates for hospitalized COVID-19 patients were 143/738 (19.4%, female) and 213/919 (23.2%, male). The corresponding numbers for COVID-19-negative individuals were 1,027/17,134 (6.0%, female) and 1,158/14,349 (8.1%, male) and for influenza-positive individuals 240/3,851 (6.2%, female) and 276/3,349 (8.2%, male). Mortality for COVID-19-positive inpatients was significantly increased in females and males compared to COVID-19-negative females (RR 3.23; 95% CI = 2.76-3.79) and males (RR 2.87; 95% CI = 2.52–3.27) and influenza-positive females (RR 3.11; 95% CI = 2.57–3.77) and males (RR 2.81; 95% CI = 2.39–3.31). See Table 2 and Supplementary Table 6 for a full outline of inpatient mortality rates stratified according to sex and age groups.

#### Mortality Rates of Outpatients Tested for COVID-19 and/or Influenza

**Figure 3:**
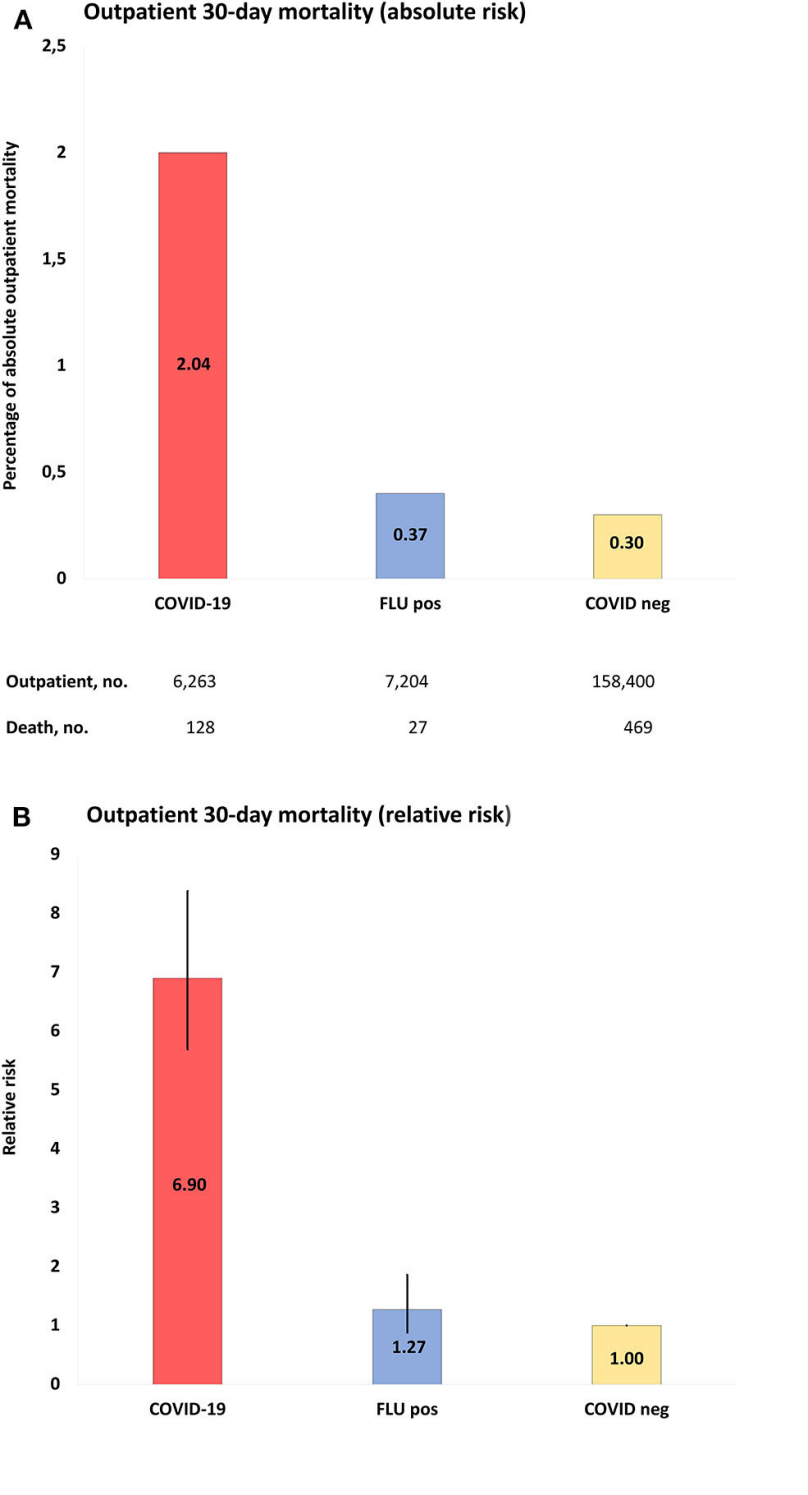
**(A)**, Absolute risk with 95% CI of COVID-19 outpatient 30-day mortality, when compared to populations of the study: COVID-19, positive COVID-19 test between March 1-June 1, 2020; FLU pos, positive influenza A/B test between November 1, 2017-March 1, 2020; COVID-neg, negative COVID-19 test between March 1-June 1, 2020. **(B)**, RR with 95% CI of outpatient mortality of study populations compared to COVID-19 negative population as reference.

Regarding outpatients, positive COVID-19 tests were associated with 128 deaths in 6,263 people (2% 30-day mortality) and negative COVID-19 tests with 469 deaths in 158,400 people (0.3%), whereas the corresponding numbers for influenza-tested people were 27/7,204 (0.4%; positive test) and 129/19,314 (0.7%; negative test). Mortality rates for COVID-19-positive outpatients were increased by RR 6.90 (95% CI = 5.69–8.38) compared to COVID-19-negative outpatients, by RR 5.45 (95% CI = 3.61–8.25) compared to influenza-positive outpatients, and by RR 3.06 (95% CI = 2.40–3.90) compared to influenza-negative outpatients. Figure 3 and Supplementary Tables 6–9 show details.

The 30-day mortality rates for outpatients with COVID-19 were 20/62,701 (0.03%), 33/1,043 (3.2%), and 92/401 (22.9%) for age groups 40–59, 60–80, and >80 years, respectively. The corresponding numbers for COVID-19 negative individuals were 161/33,166 (0.5%) and 288/7,874 (3.7%) in the age groups 60–80 and >80 years, and for influenza-positive individuals ≤5/1,761 and 20/415 (4.8%), respectively. The case numbers were too low in the remaining age groups for statistics. The 30-day mortality rates for outpatients with COVID-19 were 75/3,937 (1.9%) and 53/2,326 (2.3%) in females and males, respectively. The corresponding numbers for COVID-19 negative individuals were 261/99,512 (0.3%) and 208/58,888 (0.4%) for females and males, respectively, and for influenza-positive individuals 17/4,284 (0.4%) and 10/2,920 (0.3%), respectively. Outpatient 30-day mortality was significantly increased in COVID-19 males and females compared to COVID-19 negative and Influenza-positive and negative individuals. See Table 2 and Supplementary Table 6 for full details of outpatient mortality rates stratified according to age and sex.

### Secondary Outcomes: New-Onset Comorbidities

**Figure 4:**
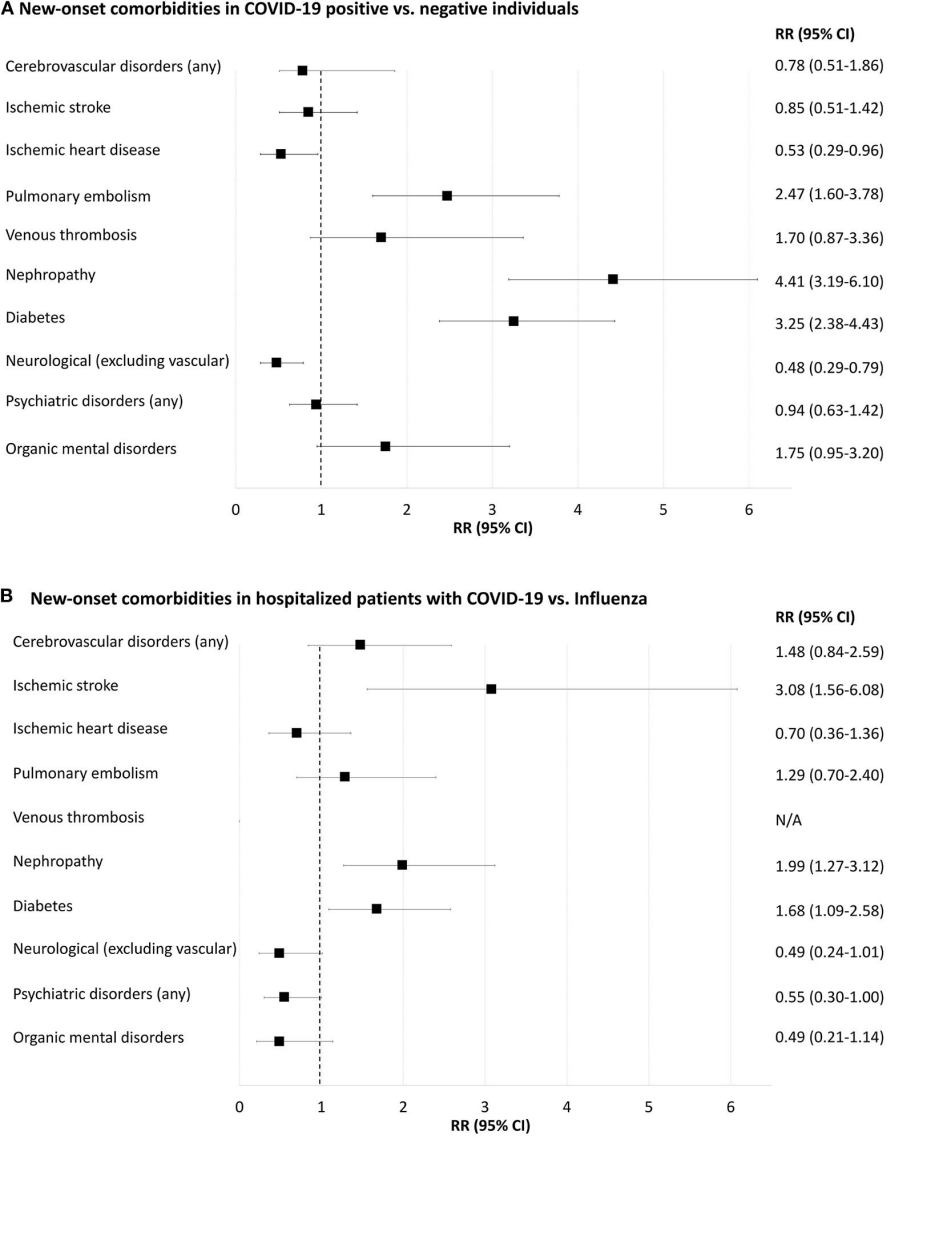
Forest-plot showing relative risk of new-onset comorbidities 30 days after positive or negative COVID-19 or influenza A/B tests (i.e., prior comorbidities excluded). **(A)**, COVID-19 positive compared to COVID-19 negative individuals. **(B)**, Inpatients with positive COVID-19 compared to inpatients with positive influenza. N/A, not applicable because of too few cases for meaningful statistics (≤ 5 individuals). New-onset delirium, neurotic and anxiety disorders, affective disorders, schizophrenia and delusional disorders and rheumatoid arthritis yielded too few cases for meaningful statistics (data shown in Supplementary Tables 10, 11).

Figure 4 and Supplementary Tables 10, 11 display data regarding novel diagnoses after COVID-19 and influenza tests.

#### New-Onset Comorbidities Among COVID-19-Positive and COVID-19-Negative Individuals

Pulmonary embolism 30 days after testing was more frequent in COVID-19-positive compared to COVID-19-negative individuals [RR 2.47 (95% CI = 1.60–3.78)], Supplementary Table 10. Diabetes and renal failure were also more frequent in COVID-19-positive compared to negative individuals (0.6 vs. 0.2% and 0.6 vs. 0.1%, respectively; both *p* < 0.001). Neurological disorders (excluding vascular disorders) and ischemic heart disease were less frequent in COVID-19-positive than in COVID-19-negative people (0.2 vs. 0.5% and 0.1 vs. 0.3%, respectively; both *p* < 0.05). Rates of new-onset cerebrovascular disorders, venous thrombosis and psychiatric disorders were not significantly different between the two populations.

#### New-Onset Comorbidities in Inpatients Tested Positive for COVID-19 vs. Influenza-Positive Individuals

Incident ischemic stroke 30 days after a test was more frequent in COVID-19-positive inpatients compared to those with influenza, RR 3.10 (95% CI = 1.56–6.08), Supplementary Table 11. New-onset diabetes and nephropathy were more frequent in COVID-19 positive compared to influenza-positive inpatients (1.9 vs. 1.2% and 1.8 vs. 0.9%, respectively; both *p* < 0.05). Rates of new-onset pulmonary embolism, neurological disorders and psychiatric disorders were not statistically different.

#### New-Onset Comorbidities in Outpatients Tested Positive for COVID-19 vs. Influenza-Positive Individuals

Incidence diagnoses 30 days after positive tests in outpatients yielded either too low numbers for meaningful statistics or were not statistically different (Supplementary Table 11).

### Sensitivity Analysis

The COVID-19-positive population was compared to a population of influenza-tested individuals from the same time period, March 1-June 1, 2020, i.e., outside the influenza peak season (FLU-19). In total, 12,502 people were tested for influenza A/B (56% inpatients; 566 positive and 8,318 negative). Inpatient mortality in FLU-19-positive and -negative populations was 26/317 (8.2%), respectively, 578/5,058 (11.4%). Inpatient mortality was significantly increased in COVID-19 compared to FLU-19-positive and -negative individuals (RR 2.62 (95% CI = 1.79–3.83), respectively, RR 1.88 (95% CI = 1.67–2.12); both *p* < 0.001).

## Discussion

To our knowledge, this is the first population-based study comparing mortality rates and new-onset comorbidities of COVID-19 patients with those of COVID-19-negative controls and individuals tested for influenza A/B. 30-day mortality was 3.0 to 6.9-fold higher in the group of individuals tested positive for COVID-19 compared to individuals tested COVID-19 negative and when compared with individuals tested for influenza. The largest difference in mortality between COVID-19 and influenza was observed in outpatients. Equally important, new-onset ischemic stroke, renal failure and diabetes occurred at increased rates in COVID-19-positive inpatients compared to influenza patients.

Previous studies have reported widely varying overall COVID-19 mortality rates, e.g., 1.4% among 1,099 cases in Wuhan, China (14), and 7.2% among 22,512 in Italy (15). In our study, the overall 30-day COVID-19 mortality was 6.1% and males over 60 years of age were overrepresented, which is well in line with previous data from COVID-19 patients from Denmark (16). Importantly, mortality rates are very different among in- and outpatients. In the COVID-19-positive inpatient population 30-day mortality was 21%, corresponding well-again with a mortality of 28% in 191 inpatients reported by Zhou et al. (17), respectively, a median 14-day mortality of 26% in 140 inpatients from Xie et al. (18). These numbers are much higher than the 2% mortality in outpatients tested COVID-19 positive (i.e., individuals from the general public not requiring hospitalization) in the present study, indicating that, not surprisingly, inpatients with COVID-19 are doing worse than outpatients.

Compared to individuals tested positive for influenza and individuals tested COVID-19-negative, COVID-19 30-day mortality was increased 3.0 to 3.10-fold for inpatients and 5.5 to 6.9-fold for outpatients. This is somewhat in contrast with an estimated 20-fold mean increase of COVID-19 mortality compared to influenza, based on indirect estimated numbers from the general public in the US (19). This discrepancy could be explained by the higher proportion of sick individuals in our influenza tested populations, as the influenza testing in Denmark is primarily done on individuals at risk due to chronic conditions. If testing for influenza A/B in Denmark had been equally widespread as for COVID-19, the excess COVID-19 mortality gap would likely have been even larger.

Thromboembolic complications in COVID-19 are assumed to be frequent (20). New-onset ischemic stroke was indeed more frequent in COVID-19 than in influenza inpatients. Increased rates of ischemic stroke in COVID-19 compared to influenza were also found in another study based on retrospective medical charts review from 2 academic centers in New York (21). Given that signs and symptoms of stroke – especially minor stroke – may be obscured by systemic illness as well as sedation and ventilation, the true risk may even be higher than the 3- to 7-fold increase reported here and in the cited work (21). We also found that the risk of new-onset diabetes was 3-fold elevated in COVID-19-positive individuals compared to negative controls and 2-fold elevated compared to influenza-positive patients. These results substantiate concerns of diabetogenic effects of COVID-19 (22), including the possibility of ketoacidosis (23). Similarly, nephropathy was frequent in our COVID-19 population, and renal failure may lead to more complications and higher in-hospital mortality (24). Ischemic heart disease appeared equally prevalent in inpatients with COVID-19 and those with influenza. Finally, pulmonary embolism occurred more often in our COVID-19 positive population compared to negative controls (albeit not compared to influenza populations).

All these comorbidities, alone or in combination, may put patients with COVID-19 at risk for multiorgan failure. This, together with hypoxemia owing to pulmonary changes, including diffuse alveolar damage with fibrin membranes, thickened alveolar walls, lymphocytic infiltration (25), and pulmonary thrombosis (25), complicated by cardiac arrhythmias, hypotensive shock (26), and possibly brainstem dysfunction (27), is being proposed as the final pathway to death in COVID-19 (28), Many of these mechanisms are unlikely to be specific enough to be reliably captured by diagnostic coding in EHR-based studies such as ours. Large prospective multicenter registries and autopsy studies comparing COVID-19 patients with COVID-19-negative controls and influenza victims are required to dissect the exact contribution of each of these factors.

Concerns for neurological and psychiatric complications in COVID-19 are increasingly being raised (11). Yet, most [albeit not all (10)] reports have revealed a predominance of relatively unspecific symptoms such as altered mental state in highly selected groups without control groups (11, 29, 30), while we report on EHR-registered diagnoses. Our results show decreased or similar frequencies of new-onset neurological and psychiatric diagnoses in COVID-19 individuals within 30 days of testing compared to influenza, which suggests either that these complications in COVID-19 are no more frequent than for severe influenza or that the nationwide lockdown in Denmark resulted in fewer contacts to the health care system by people with COVID-19 but relatively mild comorbid symptoms, including neurological and psychiatric ones. Indeed, observations from California, Italy and Denmark (31–33) indicate a lower incidence of hospitalization of patients with e.g., cardiac disease during the COVID-19 lockdown. Further, mild cognitive and emotional symptoms are not likely to be reported within 30 days, and thus the potential long-term consequences of COVID-19 could not be investigated in this study, where we investigated the acute short-term comorbidities.

### Strengths and Limitations

Strengths of our study are related, among others, to the large population numbers and the catchment area-based approach. The extracted general mortality data during years 2018–2020 corresponded well to Danish statistics mortality data (Supplementary Table 2). Numbers of COVID-19 and influenza tests, test results, admissions, and mortality rates in this study were equally consistent with the official Danish numbers (34) Further, test results of SARS-CoV-2 and influenza swabs are synchronized with the Danish national microbiology database (13), which is again linked with nationwide mortality data. Therefore, the mortality outcome can be considered virtually complete. We validated our data extraction strategy by ensuring that two individual searches supervised by two independent Epic Slicer-Dicer experts yielded identical results.

As to limitations, we were unable to adjust for confounding factors such as socioeconomics, lifestyle, ethnicity and comorbidities, owing to the use of aggregated EHR data. Instead, we performed stratified analyses according to age- and sex-groups. Of note, people who died of COVID-19 without being tested (i.e., without being recognized as COVID-19 victims) were for obvious reasons not included in our results, which might have led to an underestimation of COVID-19 mortality. Further, we could not adjust for influenza immune prophylaxis given to 10–15% of the Danish population annually, primarily patients in at risk-groups due to chronic conditions (35). Vaccination reduces influenza rates and increases the chance of a milder course of influenza, leading to a lower probability of new-onset comorbidities and decreased mortality. These effects depend on the effectiveness of the vaccine which varies each year and was particularly low during the 2017/2018 season owing to a mismatch between strains used in the production of the vaccine and those causing the seasonal epidemic (36). In the 2017/2018 peak influenza season, influenza B was identified in 68% patients tested positive for influenza, while only 17% were tested positive for influenza A (H3N2) and 14% for influenza A (H1N1). Vaccine effectiveness was 30–33%, 0–13%, and 45–50% for influenza B, A (H1N1) and A (H3N2), respectively (36). When comparing COVID-19 with influenza A/B, it must thus be kept in mind that influenza vaccination likely has had a decreasing effect on overall morbidity and mortality in the influenza population. Selection bias might also be considerable because individuals were tested in hospital settings (even as outpatients), and the testing strategy of COVID-19 in Denmark has been much more comprehensive compared to influenza. Furthermore, we only investigated the individuals tested for COVID-19 or influenza, whereas if instead comparing with the entire background population in the capture area, the mortality and morbidity ratios would likely be more increased as the tested population likely have more symptoms and comorbidities than the population not tested.

## Conclusions

In this first population-based study comparing individuals with COVID-19 positive test results with individuals tested negative for COVID-19 and individuals with influenza, COVID-19 was associated with substantially higher mortality. Due to use of aggregated data with limited ability to adjust for confounders, results must be interpreted with caution, but this mortality is likely even higher than the stated 3.0 to 5.5-fold increase owing to more extensive testing for COVID-19. In addition, we observed higher rates of new-onset ischemic stroke, diabetes and renal failure. Next, middle- and long-term follow-up data are required to investigate mortality trajectories in COVID-19 vs. influenza populations, and molecular and genetic studies will have to elucidate the specific biological mechanisms behind COVID-19's higher mortality and morbidity compared to influenza.

## Data Availability Statement

The datasets presented in this study can be found in online repositories. The names of the repository/repositories and accession number(s) can be found in the article/Supplementary Material.

## Author Contributions

DK and MB contributed to the conception, design of the study, and contributed equally as senior authors. VN and MA contributed equally as first authors. All authors contributed to the intellectual conception, revision of important intellectual content, and approval of the final version of this manuscript.

## Conflict of Interest

The authors declare that the research was conducted in the absence of any commercial or financial relationships that could be construed as a potential conflict of interest.
