# Supplementary material for: Thirty-Day Mortality and Morbidity in COVID-19 Positive vs. COVID-19 Negative Individuals and vs. Individuals Tested for Influenza A/B: A Population-Based Study
Source: Front Med (Lausanne). 2020 Nov 30;7:598272. doi: 10.3389/fmed.2020.598272 (PMC7734212; doi:10.3389/fmed.2020.598272)
Supplement: Supplementary file 1 [file Data_Sheet_1.PDF]

Supplementary Appendix

Contents

1. Supplemental eTables 1-11

eTables 1-11

eTable 1. Detailed search string utilized in EPIC SLICER DICER		
**POPULATIONS**	**SEARCH METHOD**	**TIME PERIOD**
COVID-19		
Positive	All patients ≥ 18 years **AND** (Laboratory findings of Positive SARS-COV-2 test **OR** CORONAVIRUS 2019-NCOV)	March 1 – June 1, 2020
Negative	All patients ≥ 18 years **AND** (Laboratory findings of Negative SARS-COV-2 test **OR** CORONAVIRUS 2019-NCOV) **AND** (exclusion of positive SARS-COV-2 test OR CORONAVIRUS 2019-NCOV)	March 1 – June 1, 2020
Influenza A/B		
Positive, FLU	All patients ≥ 18 years **AND** (Positive laboratory findings of, INFLUENZA A, INFLUENZA B, INFLUENZA A RNA, INFLUENZA B RNA, INFLUENZA VIRUS B, INFLUENZA TYPE A, INFLUENZA TYPE B, INFLUENZA A RNA (POC) **OR** INFLUENZA B RNA (POC))	November 1, 2017 – March 1, 2020
Positive, FLU17	All patients ≥ 18 years **AND** (Positive laboratory findings of, INFLUENZA A, INFLUENZA B, INFLUENZA A RNA, INFLUENZA B RNA, INFLUENZA VIRUS B, INFLUENZA TYPE A, INFLUENZA TYPE B, INFLUENZA A RNA (POC) **OR** INFLUENZA B RNA (POC))	November 1, 2017 – March 1, 2018
Positive, FLU18	All patients ≥ 18 years **AND** (Positive laboratory findings of, INFLUENZA A, INFLUENZA B, INFLUENZA A RNA, INFLUENZA B RNA, INFLUENZA VIRUS B, INFLUENZA TYPE A, INFLUENZA TYPE B, INFLUENZA A RNA (POC) **OR** INFLUENZA B RNA (POC))	November 1, 2018 – March 1, 2019
Positive, FLU19	All patients ≥ 18 years **AND** (Positive laboratory findings of, INFLUENZA A, INFLUENZA B, INFLUENZA A RNA, INFLUENZA B RNA, INFLUENZA VIRUS B, INFLUENZA TYPE A, INFLUENZA TYPE B, INFLUENZA A RNA (POC) **OR** INFLUENZA B RNA (POC)) **AND** (exclusion of Positive laboratory findings of SARS-COV-2 test **OR** CORONAVIRUS 2019-NCOV)	March 1 – June 1, 2020
Negative, FLU	All patients ≥ 18 years **AND** (Negative laboratory findings of, INFLUENZA A, INFLUENZA B, INFLUENZA A RNA, INFLUENZA B RNA, INFLUENZA VIRUS B, INFLUENZA TYPE A, INFLUENZA TYPE B, INFLUENZA A RNA (POC) **OR** INFLUENZA B RNA (POC)) **AND** (exclusion of Positive laboratory findings of, INFLUENZA A, INFLUENZA B, INFLUENZA A RNA, INFLUENZA B RNA, INFLUENZA VIRUS B, INFLUENZA TYPE A, INFLUENZA TYPE B, INFLUENZA A RNA (POC) **OR** INFLUENZA B RNA (POC))	November 1, 2017 – March 1, 2020
Negative, FLU17	All patients ≥ 18 years **AND** (Negative laboratory findings of, INFLUENZA A, INFLUENZA B, INFLUENZA A RNA, INFLUENZA B RNA, INFLUENZA VIRUS B, INFLUENZA TYPE A, INFLUENZA TYPE B, INFLUENZA A RNA (POC) **OR** INFLUENZA B RNA (POC)) **AND** (exclusion of Positive laboratory findings of, INFLUENZA A, INFLUENZA B, INFLUENZA A RNA, INFLUENZA B RNA, INFLUENZA VIRUS B, INFLUENZA TYPE A, INFLUENZA TYPE B, INFLUENZA A RNA (POC) **OR** INFLUENZA B RNA (POC))	November 1, 2017 – March 1, 2018
Negative, FLU18	All patients ≥ 18 years **AND** (Negative laboratory findings of, INFLUENZA A, INFLUENZA B, INFLUENZA A RNA, INFLUENZA B RNA, INFLUENZA VIRUS B, INFLUENZA TYPE A, INFLUENZA TYPE B, INFLUENZA A RNA (POC) **OR** INFLUENZA B RNA (POC)) **AND** (exclusion of Positive laboratory findings of, INFLUENZA A, INFLUENZA B, INFLUENZA A RNA, INFLUENZA B RNA, INFLUENZA VIRUS B, INFLUENZA TYPE A, INFLUENZA TYPE B, INFLUENZA A RNA (POC) **OR** INFLUENZA B RNA (POC))	November 1, 2018 – March 1, 2019
Negative, FLU19	All patients ≥ 18 years **AND** (Negative laboratory findings of, INFLUENZA A, INFLUENZA B, INFLUENZA A RNA, INFLUENZA B RNA, INFLUENZA VIRUS B, INFLUENZA TYPE A, INFLUENZA TYPE B, INFLUENZA A RNA (POC) **OR** INFLUENZA B RNA (POC)) **AND** (exclusion of Positive laboratory findings of, INFLUENZA A, INFLUENZA B, INFLUENZA A RNA, INFLUENZA B RNA, INFLUENZA VIRUS B, INFLUENZA TYPE A, INFLUENZA TYPE B, INFLUENZA A RNA (POC) **OR** INFLUENZA B RNA (POC)) **AND** (exclusion of Positive laboratory findings of SARS-COV-2 test OR CORONAVIRUS 2019-NCOV)	March 1 – June 1, 2020
**DEMOGRAPHICS, mortality and prior/new-onset comorbidities**		
SEX		
Female / male	Use of slicing function for sex distribution of male or female on all POPULATIONS	

Age		
Mean	Use of measure function to calculate mean age on all POPULATIONS	
Range	Use of slicing function for age range on all POPULATIONS	
Admission status		
Inpatient	POPULATION as defined **PLUS** overlapping criteria of hospitalization at the same time as laboratory test is performed	
Outpatient	POPULATION as defined **AND** (exclusion of individuals with a corresponding laboratory test **PLUS** overlapping criteria of hospitalization at the same time as laboratory test is performed)	
Mortality		
	POPULATION as defined **PLUS** sequential criteria of death up to 30 days after COVID-19 or Influenza laboratory test	
Comorbidities		
Medical history prior to testing	POPULATION as defined **PLUS** sequential criteria of present diagnoses up to 20 years prior to COVID-19 or Influenza laboratory test	
New-onset comorbidities after testing	Population as defined **AND** (exclusion of sequential criteria of present diagnosis up to 20 years before performed COVID-19 or Influenza laboratory test) **PLUS** sequential criteria of diagnosis up to 30 days after COVID-19 or Influenza laboratory test	
**List of ICD-10 diagnosis codes used for identifying prior and new-onset comorbidities**		
Neurological diagnosis, any (G00-99)		
Cerebrovascular diagnosis (I60-69, G45 and G46)		
Ischemic stroke (I63)		
Psychiatric diagnosis, any (F00-99)		
Ischemic heart disease (I20-25)		
Heart failure (I50)		
Diabetes, any (E10-14)		
Chronic lower respiratory disease (J40-47)		
Obesity (E66)		
Anxiety (F40-48, neurotic, stress-related and somatoform disorders)		
depression/affective disorder (F30-39, mood (affective) disorders)		
schizophrenia (F20-29, Schizophrenia, schizotypal and delusional disorders)		
Organic mental disorders (F00-09)		
Delirium (F05)		
Pulmonary embolism (I26)		
Rheumatoid arthritis (M05-06, seropositive and other Rheumatoid arthritis)		
Nephropathy (N17-19, Acute, chronic and unspecified renal failure)		
Venous thrombosis (I80, all phlebitis and thrombophlebitis)		

Validation of mortality data

To ensure verification of data, absolute mortality rates extracted from electronic health records (EPIC) were compared with official Danish statistics numbers:

**eTable 2. Absolute mortality rates extracted from electronic health records (EPIC) and compared to official Danish numbers**		
**Year**	**EPIC**	**Official Danish statistics data**
**2018 (Jan-Dec)**	25,733	24,791
**2019 (Jan-Dec)**	25,992	24,377

EPIC-extracted general mortality data during years 2017-2019 corresponded well to Danish statistics mortality data (https://www.statistikbanken.dk/FOD507) covering the Capital (Greater Copenhagen, Bornholm) and Zealand Regions of Denmark. The discrepancy between the EPIC and official numbers is due to registration of e.g. tourists and patients from outside the Capital and Zealand Regions, treated for whatever reasons at a hospital in these 2 regions (which includes Rigshospitalet, Copenhagen University Hospital, a large tertiary referral center).

Reference: Bliddal M, Broe A, Pottegård A, Olsen J, Langhoff-Roos J. The Danish Medical Birth Register. Eur. J. Epidemiol. 2018;33(1):27–36.

eTable 3. Demographics and comorbidities in total populations (i.e. in- and outpatients combined)							
	**COVID-19 (N = 7,920)**	**COVID-neg (N=189,883)**	***OR (95% CI)***	***p value***	**FLU (N = 14,404)**	***OR (95% CI)***	***p value***
**Age – years, no. (%)**							
**Mean**	51	50			58		
**18 - 40**	2,492 (31.5)	62,115 (32.7)	0.94 (0.9-0.99)	0.02	3,322 (23.1)	1.53 (1.44-1.63)	< 0.001
**40 - 60**	2,936 (37.1)	69,491 (36.6)	1.02 (0.97-1.07)	0.39	4,050 (28.1)	1.50 (1.42-1.60)	< 0.001
**60 - 80**	1,679 (21.2)	44,427 (2.4)	0.88 (0.83-0.93)	< 0.001	4,637 (32.2)	0.57 (0.53-0.60)	< 0.001
**> 80**	813 (10.3)	13,850 (7.3)	1.45 (1.35-1.57)	< 0.001	2,395 (16.6)	0.57 (0.53-0.62)	< 0.001
**Sex (%)**							
**Women**	4,666 (58.9)	116,425 (61.3)	0.90 (0.86-0.95)	< 0.001	8,116 (56.3)	1.11 (1.05-1.17)	< 0.001
**Prior medical diagnoses - no. (%)**							
**Neurological, any**	780 (9.8)	19,072 (10.0)	0.98 (0.91-1.06)	0.57	1,499 (10.4)	0.86 (0.79-0.95)	0.002
**Cerebrovascular, any**	286 (3.6)	6,517 (3.4)	1.05 (0.93-1.19)	0.39	610 (4.2)	0.85 (0.73-0.98)	0.023
**Ischemic stroke**	163 (2.1)	4,041 (2.1)	0.97 (0.82-1.13)	0.67	295 (2.0)	1.01 (0.83-1.22)	0.05
**Psychiatric, any**	585 (7.4)	18,845 (9.9)	0.72 (0.66-0.79)	< 0.001	1,126 (7.8)	0.94 (0.85-1.04)	0.24
**Ischemic heart disease**	237 (3.0)	6,350 (3.3)	0.89 (0.78-1.02)	0.09	702 (4.9)	0.60 (0.52-0.70)	< 0.001
**Heart failure**	145 (1.8)	3,442 (1.8)	1.01 (0.85-1.19)	0.91	490 (3.4)	0.53 (0.44-0.64)	< 0.001
**Diabetes**	408 (5.2)	8,194 (4.3)	1.20 (1.09-1.33)	< 0.001	1,082 (7.5)	0.67 (0.59-0.75)	< 0.001
**Chronic lower respiratory disease**	405 (5.1)	11,877 (6.3)	0.81 (0.73-0.89)	< 0.001	1,728 (12.0)	0.40 (0.35-0.44)	< 0.001
**Obesity**	178 (2.2)	5,225 (2.8)	0.81 (0.7-0.95)	0.007	308 (2.1)	1.05 (0.87-1.27)	< 0.59

COVID-19: positive COVID-19 PCR test between March 1-June 1, 2020; FLU: positive influenza A/B PCR test between November 1, 2017 to March 1, 2020; COVID-neg: negative COVID-19 PCR test between March 1-June 1, 2020. Each patient was followed for a total of 30 days from positive test until end of follow-up or death. CI - confidence interval; no., number; OR, odds ratio. OR denotes comparisons of COVID-19 with study populations.

**eTable 4. Demographics and comorbidities in in- and outpatient populations – OR and p-values compared to COVID-19 population as exposed group**								
	**Inpatients#**				**Outpatients#**			
	**COVID-neg OR (95% CI)**	**COVID-neg p-value**	**FLU OR (95% CI)**	**FLU p-value**	**COVID-neg OR (95% CI)**	**COVID-neg p-value**	**FLU OR (95% CI)**	**FLU p-value**
**Age – years**								
**18 - 39**	0.52 (0.45-0.61)	<0.001	0.83 (0.71-0.98)	0.03	1.11 (1.05-1.17)	<0.001	1.17 (1.10-1.26)	<0.001
**40 - 59**	1.26 (1.13 - 1.41)	<0.001	1.42 (1.26-1.61)	<0.001	1.02 (0.97-1.07)	0.45	1.16 (1.08-1.24)	<0.001
**60 - 80**	1.12 (1.01-1.24)	0.03	0.94 (0.84-1.05)	0.24	0.75 (0.71-0.81)	<0.001	0.62 (0.57-0.67)	<0.001
**> 80**	1.41 (1.26-1.58)	<0.001	0.87 (0.77-0.99)	0.03	1.31 (1.18-1.45)	<0.001	1.12 (0.97-1.29)	0.12
**Sex**								
**Women**	0.68 (0.61-0.74)	<0.001	0.70 (0.63-0.78)	<0.001	1.00 (0.95-1.06)	0.95	1.16 (1.08-1.24)	<0.001
**Prior medical diagnoses - no. (%)**								
**Neurological, any**	1.05 (0.92-1.20)	0.43	1.20 (1.04-1.39)	0.01	0.86 (0.78-0.94)	0.001	1.25 (1.09-1.42)	0.001
**Cerebrovascular, any**	0.95 (0.79-1.13)	0.54	1.29 (1.06-1.57)	0.01	0.99 (0.83-1.17)	0.92	1.34 (1.05-1.71)	0.02
**Ischemic stroke incl. TIA**	0.84 (0.67-1.07)	0.16	2.11 (1.60-2.77)	<0.001	0.93 (0.75-1.16)	0.53	0.75 (0.57-0.99)	0.04
**Psychiatric, any**	0.53 (0.45-0.61)	<0.001	1.08 (0.92-1.27)	0.37	0.78 (0.70-0.86)	<0.001	1.47 (1.26-1.72)	<0.001
**Ischemic heart disease**	1.07 (0.90-1.27)	0.46	1.18 (0.98-1.43)	0.08	0.61 (0.49-0.75)	<0.001	0.66 (0.51-0.86)	0.002
**Heart failure**	1.02 (0.83-1.26)	0.84	1.03 (0.82-1.29)	0.79	0.72 (0.53-0.97)	0.03	0.76 (0.52-1.11)	0.15
**Diabetes**	1.17 (1.00-1.36)	0.04	1.05 (0.89-1.24)	0.55	1.08 (0.94-1.24)	0.28	0.94 (0.78-1.13)	0.52
**Chronic lower respiratory disease**	0.86 (0.74-1.01)	0.06	0.53 (0.45-0.62)	<0.001	0.70 (0.61-0.80)	<0.001	0.69 (0.58-0.82)	<0.001
**Obesity**	0.67 (0.49-0.90)	0.007	1.20 (0.86-1.67)	0.27	0.85 (0.71-1.01)	0.06	1.08 (0.85-1.37)	0.54
COVID-neg: negative COVID-19 PCR test between March 1-June 1, 2020. FLU: positive influenza A/B PCR test between November 1, 2017 to March 1, 2020; Each patient was followed for a total of 30 days from positive test until end of follow-up or death. TIA: Transient ischemic attack. # at the time of testing								

eTable 5. Demographics and comorbidities of patients in COVID-19 population and subgroups							
	**COVID-19 (N = 7,920)**	**FLU-19 (N = 566)**	**FLU-19 neg (N = 8,318)**	**FLU-18 (N = 3,838)**	**FLU-18 neg (N = 11,153)**	**FLU-17 (N = 4,721)**	**FLU-17 neg (N = 9,104)**
**Age – years, no. (%)**							
**mean**	51	55	60	55	59	59	61
**18 - 40**	2,492 (31.5)	164 (29.0)	1,570 (18.9)	992 (25.8)	2,388 (21.4)	900 (19.1)	1,834 (20.1)
**40 - 60**	2,936 (37.1)	145 (25.6)	2,288 (27.5)	1,215 (31.7)	2,791 (25.0)	1,367 (29.0)	2,136 (23.5)
**60 - 80**	1,679 (21.2)	170 (30.0)	2,995 (36.0)	1,111 (28.9)	3,998 (35.8)	1,590 (33.7)	3,281 (36.0)
**> 80**	813 (10.3)	87 (15.4)	1,465 (17.6)	520 (13.5)	1,976 (17.7)	864 (18.3)	1,853 (20.4)
**Sex (%)**							
**Women**	4,666 (58.9)	308 (54.4)	4,500 (54.1)	2,171 (56.6)	6,220 (55.8)	2,639 (55.9)	5,087 (55.9)
**Prior medical history - no. (%)**							
**Neurological, any**	780 (9.8)	84 (14.8)	1,364 (16.4)	453 (11.8)	1,495 (13.4)	427 (9.0)	994 (10.9)
**Cerebrovascular**	286 (3.6)	43 (7.6)	673 (8.1)	158 (4.1)	686 (6.2)	175 (3.7)	465 (5.1)
**Ischemic stroke**	163 (2.1)	26 (4.6)	379 (4.6)	68 (1.8)	367 (3.3)	84 (1.8)	218 (2.4)
**Psychiatric, any**	585 (7.4)	48 (8.5)	1,208 (14.5)	368 (9.6)	1,242 (11.1)	307 (6.5)	781 (8.6)
**Ischemic heart disease**	237 (3.0)	31 (5.5)	686 (8.2)	190 (5.0)	756 (6.8)	187 (4.0)	545 (6.0)
**Heart failure**	145 (1.8)	23 (4.1)	496 (6.0)	135 (3.5)	560 (5.0)	146 (3.1)	380 (4.2)
**Diabetes**	408 (5.2)	43 (7.6)	864 (10.4)	283 (7.4)	1,020 (9.1)	335 (7.1)	761 (8.4)
**CLRD**	405 (5.1)	73 (12.9)	1,500 (18.0)	491 (12.8)	1,865 (16.7)	525 (11.1)	1,414 (15.5)
**Obesity**	178 (2.2)	17 (3.0)	204 (2.5)	101 (2.6)	274 (2.5)	75 (1.6)	179 (2.0)
**Admission – no. (%)**							
**Inpatients**	1,657 (20.9)	317 (56.0)	5,058 (60.8)	1,842 (48.0)	6,220 (55.8)	2,376 (50.3)	5,503 (60.4)
**Outpatients**	6,263 (79.1)	249 (44.0)	3,260 (39.2)	1,996 (52.0)	4,933 (44.2)	2,345 (49.7)	3,601 (39.6)

Basic demographics and comorbidities of study populations. COVID-19, Positive COVID-19 PCR test between March 1 to June 1, 2020; FLU-19, Positive influenza PCR test between March 1 to June 1, 2020; FLU-18, Positive influenza A/B PCR test between November 1, 2018 to March 1, 2019; FLU-17, Positive influenza A/B PCR test between November 1, 2017 to March 1, 2018; COVID-neg, Negative COVID-19 PCR test between March 1 to June 1, 2020. Patients with a positive or negative test were included in the given inclusion period. Each included patient was followed for a total of 30 days from positive test until end of follow-up or death. CLRD, Chronic lower respiratory disease; no., number; COVID, coronavirus disease

eTable 6. Relative risk of mortality, in- and outpatients in main populations, 30 days after test compared to COVID-19 negative individuals

	Total inpatient N (mean age)	Death inpatient N	*Inpatient RR (95% CI)*	Total outpatient N (mean age)	Death outpatient N	*Outpatient RR (95% CI)*
**COVID-19 positive**						
**Overall**	1,657 (65)	356	3.10 (2.80-3.42)†	6,263 (47)	128	6.9 (5.69-8.38)†
**18-39 years**	182 (30)	<5	N/A	2,310 (29)	<5	N/A
**40-59 years**	427 (50)	16	1.61 (0.97-2.67)	2,509 (49)	<5	N/A
**60-80 years**	636 (71)	150	2.65 (2.27-3.08)†	1,043 (67)	33	6.52 (4.50-9.43)†
**> 80 years**	412 (86)	190	2.73 (2.42-3.08)†	401 (87)	92	6.27 (5.07-7.76)†
**COVID-19 negative**						
**Overall**	31,483 (60)	2,185	1 (ref)	158,400 (48)	469	1 (ref)
**18-39 years**	7,456 (30)	15	1 (ref)	54,659 (30)	<5	1 (ref)
**40-59 years**	6,790 (50)	158	1 (ref)	62,701 (50)	20	1 (ref)
**60-80 years**	11,261 (71)	1,004	1 (ref)	33,166 (71)	161	1 (ref)
**> 80 years**	5,976 (86)	1,008	1 (ref)	7,874 (86)	288	1 (ref)
**FLU positive**						
**Overall**	7,200 (66)	516	1.03 (0.94-1.13)	7,204 (49)	27	1.27 (0.85-1.86)
**18-39 years**	931 (29)	<5	N/A	2,390 (29)	<5	N/A
**40-59 years**	1,413 (51)	26	0.79 (0.52-1.19)	2,638 (49)	<5	N/A
**60-80 years**	2,876 (71)	214	0.83 (0.72-0.96)‡	1,761 (69)	<5	N/A
**> 80 years**	1,980 (87)	271	0.81 (0.72-0.92)‡	415 (85)	20	1.32 (0.85-2.05)
**FLU negative**						
**Overall**	26,366 (65)	2,873	1.57 (1.49-1.66)†	19,314 (50)	129	2.26 (1.86-2.74)†
**18-39 years**	3,867 (29)	40	5.14 (2.84-9.30)†	6,664 (29)	<5	N/A
**40-59 years**	4,757 (51)	224	2.02 (1.66-2.47)†	6,454 (49)	6	2.91 (1.17-7.25)‡
**60-80 years**	10,939 (71)	1,307	1.34 (1.24-1.45)†	4,963 (69)	51	2.12 (1.55-2.90)†
**> 80 years**	6,803 (86)	1,302	1.13 (1.05-1.22)†	1,233 (85)	71	1.57 (1.22-2.03)†

Relative mortality rates divided in inpatients and outpatients. COVID-19 negative individuals were compared as the reference group with all other study populations. COVID-19, positive COVID-19 test between March 1-June 1, 2020; FLU positive, positive influenza A / B test between November 1, 2017-March 1, 2020; FLU negative, negative influenza A/B test between November 1, 2017-March 1, 2020; COVID-neg, negative COVID-19 test between March 1-June 1, 2020. Each included patient was followed for a total of 30 days from positive test until end of follow-up or death. ‡ $p < 0.05$; † $p < 0.001$; N/A, not applicable; ref, reference group

eTable 7. Mortality rates, 30 days after test in subgroups*

	Total population	Inpatients		Outpatients	
	N	N (%)	Death (%)	N (%)	Death (%)
**COVID-19 positive**	7,920	1,657 (20.9)	356 (21.5)	6,263 (79.1)	128 (2.0)
**COVID-19 negative**	189,883	31,483 (16.6)	2,185 (1.4)	158,400 (83.4)	469 (0.3)
**FLU positive**	14,404	7,200 (50.0)	516 (7.2)	7,204 (50.0)	27 (0.4)
**FLU negative**	45,680	26,366 (57.7)	2,873 (11)	19,314 (42.3)	129 (0.7)
**FLU-19 positive**	566	317 (56.0)	26 (8.2)	249 (44.0)	≤5
**FLU-19 negative**	8,318	5,058 (60.8)	578 (11.4)	3,260 (39.2)	74 (2.3)
**FLU-18 positive**	3,838	1,842 (48.0)	121 (6.6)	1,996 (52.0)	9 (0.5)
**FLU-18 negative**	11,153	6,220 (55.8)	649 (10.4)	4,933 (44.2)	41 (0.8)
**FLU-17 positive**	4,721	2,376 (50.3)	174 (7.3)	2,345 (49.7)	≤5
**FLU-17 negative**	9,104	5,503 (60.4)	597 (10.8)	3,601 (39.6)	21 (0.6)

Absolute mortality rates of study populations divided in inpatients and outpatients. COVID-19, positive COVID-19 test between March 1-June 1, 2020; FLU-18, positive influenza A/B test between November 1, 2018-March 1, 2019; FLU-17, positive influenza A/B test between November 1, 2017-March 1, 2018; COVID-neg, negative COVID-19 test between March 1-June 1, 2020. Each included patient was followed for 30 days from positive test until end of follow-up or death.* Data presented as ≤5 is due to the General Data Protection Regulation law restricting display of results with 5 or fewer patients, thus no statistical analysis was performed.

eTable 8. Relative risk of mortality, in- and outpatients in subgroups, 30 days after test compared to COVID-19 positive individuals as exposed group

Pairwise comparisons	Inpatient, RR (95% CI)	Outpatient, RR (95% CI)
**COVID-19-positive**	3.10 (2.80-3.42)†	6.90 (5.69-8.38)†
**COVID-19-negative**	1 (ref)	1 (ref)
**COVID-19-positive**	2.62 (1.79-3.83)†	N/A
**FLU-19-positive**	1 (ref)	N/A
**COVID-19-positive**	2.62 (1.79-3.83)†	0.90 (0.68-1.20)
**FLU-19-negative**	1 (ref)	1 (ref)
**COVID-19-positive**	3.27 (2.69-3.98)†	4.53 (2.31-8.89)†
**FLU-18-positive**	1 (ref)	1 (ref)
**COVID-19-positive**	2.06 (1.83-2.32)†	2.46 (17.3-3.49)†
**FLU-18-negative**		
**COVID-19-positive**	2.93 (2.47-3.48)†	N/A
**FLU-17-positive**	1 (ref)	N/A
**COVID-19-positive**	1.98 (1.76-2.23)†	3.50 (2.21-5.55)†
**FLU-17-negative**	1 (ref)	1 (ref)

Relative mortality rates divided in inpatients and outpatients. COVID-19 positive individuals were compared as the exposed group with all other populations. COVID-19, positive COVID-19 test between March 1-June 1, 2020; FLU-18, positive influenza A/B test between November 1, 2018-March 1, 2019; FLU-17, positive influenza A/B test between November 1, 2017-March 1, 2018; COVID-neg, negative COVID-19 test between March 1-June 1, 2020. Each included patient was followed for 30 days from positive test until end of follow-up or death. N/A, not applicable. † $p < 0.001$

eTable 9. Relative risk of mortality, in- and outpatients, 30 days after test compared to COVID-19 negative individuals as reference group

Study populations	Inpatient, RR (95% CI)	Outpatient, RR (95% CI)
**COVID-19 negative**	1 (ref)	1 (ref)
**COVID-19 positive**	3.10 (2.80-3.42)†	6.9 (5.69-8.38)†
**FLU-19 positive**	1.18 (0.82-1.71)	N/A
**FLU-19 negative**	1.65 (1.51-1.80)†	7.67 (6.01-9.77)†
**FLU-18 positive**	0.95 (0.79-1.13)	1.52 (0.79-2.94)
**FLU-18 negative**	1.50 (1.38-1.63)†	2.81 (2.04-3.76)†
**FLU-17 positive**	1.06 (0.91-1.22)	N/A
**FLU-17 negative**	1.56 (1.43-1.70)†	1.96 (1.27-3.05)‡

Relative mortality rates divided in inpatients and outpatients. COVID-19 negative individuals were compared as the reference group with all other study populations. COVID-19, positive COVID-19 test between March 1-June 1, 2020; FLU-18, positive influenza A / B test between November 1, 2018-March 1, 2019; FLU-17, positive influenza A/B test between November 1, 2017-March 1, 2018; COVID-neg, negative COVID-19 test between March 1-June 1, 2020. Each included patient was followed for a total of 30 days from positive test until end of follow-up or death. ‡ $p < 0.01$; † $p < 0.001$; N/A, not applicable

eTable 10. New-onset diagnosis, 30-days after COVID-19 test*

	COVID-19 (N = 7,920)	COVID-neg (N=189,883)	*RR (95% CI)*	*p value*
New diagnosis after 30 days - no. (%)[#]				
**Cerebrovascular disorders (any)**	22 (0.3)	681 (0.4)	0.78 (0.51-1.86)	0,2409
**Ischemic stroke**	15 (0.2)	423 (0.2)	0.85 (0.51-1.42)	0,5356
**Ischemic heart disease**	11 (0.1)	497 (0.3)	0.53 (0.29-0.96)	0.0364
**Pulmonary embolism**	23 (0.3)	224 (0.1)	2.47 (1.60-3.78)	<0.0001
**Venous thrombosis**	9 (0.1)	126 (0.1)	1.71 (0.87-3.36)	0.1194
**Nephropathy**	43 (0.6)	235 (0.1)	4.41 (3.19-6.10)	<0.0001
**Diabetes**	45 (0.6)	335 (0.2)	3.25 (2.38-4.43)	<0.0001
**Rheumatoid arthritis**	≤5	18 (0.01)	N/A	N/A
**Neurological disorders (excluding vascular)**	16 (0.2)	799 (0.5)	0.48 (0.29-0.79)	0,0035
**Psychiatric disorders (any)**	24 (0.3)	593 (0.3)	0.94 (0.63-1.42)	0,7806
**Organic mental disorders**	11 (0.1)	152 (0.1)	1.75 (0.95-3.23)	0,0719
**Delirium**	≤5	91 (0.05)	N/A	N/A
**Neurotic and anxiety disorders**	≤5	211 (0.1)	N/A	N/A
**Affective disorders**	≤5	145 (0.1)	N/A	N/A
**Schizophrenia and delusional disorders**	≤5	84 (0.04)	N/A	N/A

New-onset diagnoses in all COVID-19 and influenza A/B positive and negative individuals, 30 days after testing. RR denotes relative risk (RR) of new-onset diagnoses comparisons of COVID-19 positive with COVID-19 negative individuals. COVID-19, positive COVID-19 test between March 1-June 1, 2020; COVID-neg, negative COVID-19 test between March 1-June 1, 2020. Patients were followed for 30 days from positive test until end of follow-up or death. * Data presented as ≤5 is due to the General Data Protection Regulation law restricting display of results with 5 or fewer patients, thus no statistical analysis was performed. # Percentage is calculated as new-onset diagnosis divided by sum of population excluding individuals with prior history of the investigated diagnosis.

eTable 11. New-onset diagnosis in inpatients and outpatients, 30-days after positive COVID-19 or influenza A/B test*

	Inpatients				Outpatients			
	COVID-19 (N =1,657)	Influenza (N = 7,200)	*RR (95% CI)*	*p value*	COVID-19 (N =6,263)	Influenza (N = 7,204)	*RR (95% CI)*	*p value*
New diagnosis after 30 days - no. (%)[#]								
**Cerebrovascular disorders (any)**	16 (1.0)	48 (0.7)	1.48 (0.84-2.59)	0.18	6 (0.1)	6 (0.1)	1.16 (0.37-3.59)	0.80
**Ischemic stroke**	14 (0.9)	20 (0.3)	3.08 (1.56-6.08)	0.001	≤5	≤5	N/A	N/A
**Ischemic heart disease**	10 (0.7)	63 (1.0)	0.70 (0.36-1.36)	0.29	≤5	≤5	N/A	N/A
**Pulmonary embolism**	13 (0.8)	44 (0.6)	1.29 (0.70-2.40)	0.41	10 (0.2)	6 (0.1)	1.92 (0.70-5.28)	0.21
**Venous thrombosis**	≤5	9 (0.1)	N/A	N/A	≤5	≤5	N/A	N/A
**Nephropathy**	27 (1.8)	60 (0.9)	1.99 (1.27-3.12)	0.003	16 (0.3)	≤5	N/A	N/A
**Diabetes**	28 (1.9)	73 (1.15)	1.68 (1.09-2.58)	0.019	17 (0.3)	11 (0.2)	1.77 (0.83-3.78)	0.14
**Rheumatoid arthritis**	≤5	≤5	N/A	N/A	≤5	≤5	N/A	N/A
**Neurological disorders (excluding vascular)**	8 (0.6)	73 (1.2)	0.49 (0.24-1.01)	0.054	8 (0.1)	16 (0.2)	0.58 (0.25-1.36)	0.21
**Psychiatric disorders (any)**	12 (0.8)	96 (1.5)	0.55 (0.30-1.00)	0.049	12 (0.2)	14 (0.2)	1.01 (0.47-2.17)	0.99
**Organic mental disorders**	6 (0.4)	54 (0.8)	0.49 (0.21-1.14)	0.098	≤5	≤5	N/A	N/A
**Delirium**	≤5	26 (0.4)	N/A	N/A	≤5	≤5	N/A	N/A
**Neurotic and anxiety disorders**	≤5	20 (0.3)	N/A	N/A	≤5	6 (0.1)	N/A	N/A
**Affective disorders**	≤5	21 (0.3)	N/A	N/A	≤5	≤5	N/A	N/A
**Schizophrenia and delusional disorders**	≤5	≤5	N/A	N/A	≤5	≤5	N/A	N/A

New-onset diagnoses in inpatient and outpatient COVID-19 and influenza A/B positive individuals, 30 days after testing. RR denotes relative risk (RR) of new-onset diagnoses comparisons of COVID-19 positive inpatients with influenza-positive inpatients. COVID-19, positive COVID-19 test between March 1-June 1, 2020; Influenza, positive influenza A/B test between November 1, 2017-March 1, 2020. Patients were followed for 30 days from positive test until end of follow-up or death. * Data presented as ≤5 is due to the General Data

Protection Regulation law restricting display of results with 5 or fewer patients, thus no statistical analysis was performed. # Percentage is calculated as new-onset diagnosis divided by sum of population excluding individuals with prior history of the investigated diagnosis.
